# Safety, Efficacy, and Pharmacokinetics of Combination SARS-CoV-2 Neutralizing Monoclonal Antibodies BMS-986414 (C135-LS) and BMS-986413 (C144-LS) Administered Subcutaneously in Non-Hospitalized Persons with COVID-19 in a Phase 2 Trial

**DOI:** 10.20411/pai.v9i1.660

**Published:** 2024-05-06

**Authors:** Katya C. Corado, Kara W. Chew, Mark J. Giganti, Ying Mu, Courtney V. Fletcher, Judith S. Currier, Eric S. Daar, David A. Wohl, Jonathan Z. Li, Carlee B. Moser, Justin Ritz, Arzhang Cyrus Javan, Gene Neytman, Marina Caskey, Michael D. Hughes, Davey M. Smith, Joseph J. Eron, ACTIV-2/A5401 Study Team

**Affiliations:** 1 Lundquist Institute at Harbor-UCLA Medical Center, Torrance, California; 2 Department of Medicine, David Geﬀen School of Medicine at University of California, Los Angeles, California; 3 Harvard T.H. Chan School of Public Health, Boston, Massachusetts; 4 UNMC Center for Drug Discovery, University of Nebraska Medical Center, Omaha, Nebraska; 5 Department of Medicine, University of North Carolina at Chapel Hill School of Medicine, Chapel Hill, North Carolina; 6 Department of Medicine, Brigham and Women's Hospital, Harvard Medical School, Cambridge, Massachusetts; 7 National Institutes of Health, Rockville, Maryland; 8 Quantum Clinical Trials, Miami, Florida; 9 Laboratory of Molecular Immunology, The Rockefeller University, New York, New York; 10 University of California, San Diego, California

**Keywords:** COVID-19, SARS-CoV-2, monoclonal antibodies, outpatient treatment, subcutaneous

## Abstract

**Background:**

Outpatient COVID-19 monoclonal antibody (mAb) treatment via subcutaneous delivery, if eﬀective, overcomes the logistical burdens of intravenous administration.

**Methods:**

ACTIV-2/A5401 was a randomized, masked placebo-controlled platform trial where participants with COVID-19 at low risk for progression were randomized 1:1 to subcutaneously administered BMS-986414 (C135-LS) 200 mg, plus BMS-986413 (C144-LS) 200 mg, (BMS mAbs), or placebo. Coprimary outcomes were time to symptom improvement through 28 days; nasopharyngeal SARS-CoV-2 RNA below the lower limit of quantification (LLoQ) on days 3, 7, or 14; and treatment-emergent grade 3 or higher adverse events (TEAEs) through 28 days.

**Results:**

A total of 211 participants (105 BMS mAbs and 106 placebo) initiated study product. Time to symptom improvement favored the active therapy but was not significant (median 8 vs 10 days, *P*=0.19). There was no significant diﬀerence in the proportion with SARS-CoV-2 RNA <LLoQ at day 3 (risk ratio [RR] for BMS mAbs versus placebo: 1.03; 95%CI: 0.80, 1.32), at day 7 (RR: 1.04; 95%CI: 0.94, 1.15), or at day 14 (RR: 1.00; 95%CI: 0.90, 1.12). Fewer grade 3 TEAEs were reported for the BMS mAbs arm than placebo (RR: 0.58 [95%CI: 0.25, 1.32]). Through day 28, there were no deaths, and there were 4 hospitalizations in the BMS mAbs arm versus 3 in the placebo arm. Higher early plasma mAb concentrations were associated with more favorable outcomes.

**Conclusions:**

While safe, the BMS mAbs delivered subcutaneously were not eﬀective at treating COVID-19 at low risk for progression. The lack of clinically significant activity may relate to the pharmacokinetics of subcutaneous administration of mAbs.

## INTRODUCTION

The coronavirus disease 2019 (COVID-19) pandemic demanded the timely development of new therapeutics to address the global health crisis, and due to their specificity and safety profile, anti-SARS-CoV-2 monoclonal antibodies (mAbs) became a powerful tool to combat the early pandemic. Several mAbs were utilized early in the pandemic under Emergency Use Authorization (EUA) for outpatient treatment of COVID-19, including bamlanivimab/etesevimab, casirivimab/imdevimab, sotrovimab and bebtelovimab [1]. Delivery of these mAbs was primarily via intravenous (IV) infusions, and those with alternative modes of delivery (casirivimab/imdevimab and sotrovimab) did so with limited clinical data to support these routes of administration [2–6]. Delivery of mAbs to the general population had numerous challenges including logistical hurdles of opening infusion centers for persons with infectious COVID-19, racial disparities in delivery of mAbs, particularly in Latinx and Black communities [7, 8], and difficulty in accessing infusion centers for rural populations [9] and those in nursing homes [10]. Thus, a more convenient, less resource-intensive alternative to infusion delivery continues to be needed, particularly considering that mAbs continue to be developed for COVID-19 treatment.

BMS-986414 (C135-LS) and BMS-986413 (C144-LS) were developed as recombinant, fully human mAbs of the IgG1κ and λ isotope from 2 individuals who recovered from COVID-19. The mAbs bind SARS-CoV-2 spike protein RBD at non-overlapping sites [11] and were Fc-modified to extend their half-lives in humans [12]. *In vitro* neutralization assays showed that both antibodies demonstrated neutralizing activity against authentic SARS-CoV-2 with IC_50_ of 3.0 and 2.6 ng/mL and IC_90_ of 10.43 and 21.68 ng/mL, respectively. *In vitro* selection of viral escape variants that emerged with the use of BMS-986413 (E48K and Q493R) and BMS-986413 (R346S and N440K) individually was overcome when used in combination [12]. *In vitro* neutralization IC_50_ against the Delta variant for BMS-986414 and BMS-986413 were 2.8 ng/mL and 2.2 ng/mL, respectively (Christian Gaebler, MD, MSc, electronic communication, April 18, 2023).

Phase 1 evaluation of both IV and subcutaneous (SC) administration of BMS-986414 (C135-LS) and BMS-986413 (C144-LS) [12, 13] supported further clinical evaluation of a 200 mg SC dose of each mAb. Here, we report the safety as well as antiviral and clinical efficacy of a combined 400 mg SC dose (200 mg BMS-986414 [C135-LS] and 200 mg BMS-986413 [C144-LS]), henceforth referred to as the BMS mAbs, for treatment of mild-to-moderate COVID-19 in non-hospitalized adults within the ACTIV-2/A5401 platform trial.

## METHODS

### Trial Design

ACTIV-2/A5401 was a multicenter, phase 2/3, outpatient, adaptive platform randomized controlled trial for the evaluation of therapeutics for mild-to-moderate COVID-19. The protocol was approved by a central institutional review board (IRB), Advarra, with additional local IRB approval if required by participating sites. All participants provided informed consent. Additional details of the trial design and evaluation of other agents have been published elsewhere [14–19].

### Study Population

The study population consisted of non-hospitalized adults (18 years of age) with confirmed SARS-CoV-2 infection, as determined by U.S. Food and Drug Administration (FDA)-authorized PCR or antigen test from a sample collected within 10 days prior to study entry. Participants were enrolled ≤ 7 days from self-reported onset of COVID-19-related symptoms and had one or more symptoms present within 24 hours prior to study entry. Enrollment was limited to individuals at lower risk for progression (Supplemental Table 1) to severe outcomes because several mAbs had received FDA EUA and were available for treatment of mild-to-moderate COVID-19 in patients at high risk for progression to hospitalization or death. Participants who were pregnant or currently breastfeeding, with oxygen saturation < 92%, or skin conditions that compromised the safety or assessment of SC injections were excluded.

### Patient Consent

The protocol was approved by a central IRB, Advarra (Pro00045266), with additional local IRB review and approval as required by participating sites. All participants provided written informed consent.

### Randomization and Study Intervention

All participants underwent a 2-step randomization [20]. The first randomization occurred with equal probability to groups corresponding to each of the investigational agents under study for which the participant was eligible. The second randomization was within each of these groups to an active agent versus placebo with a randomization of r:1, where r is the number of groups in the first randomization. Randomization was stratified by duration of symptoms prior to enrollment (≤ 5 days or > 5 days).

A control group was constructed for analysis, pooling participants who were eligible for the BMS mAbs but were randomized to a placebo (placebo for the BMS mAbs or placebo for other concurrently enrolling agents).

Participants randomized to the BMS mAbs received 4 SC injections (total dose of 400 mg) at study entry in the abdomen, upper arms, and/or thighs. This included 2 administrations of 100 mg BMS-986414 (C135-LS) SC on the leﬅ side for a total of 200 mg, and 2 administrations of 100 mg BMS-986413 (C144-LS) SC on the right side for a total of 200 mg. A diagram of approved locations was provided. There was no diﬀerence expected based on injection site. The instructions were identical for active and placebo study products. Preparation of the vials was done by an un-blinded pharmacist. Injections were administered by masked study staﬀ.

### Study Procedures

In-person clinical assessments on days 0 (day of initiation of study product), 3, 7, 14, and 28 included safety evaluations, oxygen saturation measurements, and collection of pharmacokinetic (PK) samples and nasopharyngeal (NP) swabs collected by research staﬀ. Plasma samples for PK analysis were also collected at weeks 12, 24, 48, and 72. Forty participants who received the BMS mAbs or its placebo had an additional visit on day 1 to obtain samples for PK analysis.

Treatment-emergent adverse events (TEAEs) of special interest (AESIs) included grade ≥ 3 injection-related reactions within 72 hours of administration and grade ≥ 1 allergic or hypersensitivity reactions within 24 hours of administration deemed related to the study product by site investigators.

Participants completed a daily diary from day 0 (prior to starting the study product) through day 28, including a self-report of whether they had returned to their usual (pre-COVID-19) health, and the severity of 13 targeted COVID-19 symptoms, self-reported as absent, mild, moderate, or severe. The targeted symptoms were fever or feeling feverish, cough, shortness of breath or difficulty breathing at rest or with activity, sore throat, body pain or muscle pain/aches, fatigue, headache, chills, nasal obstruction or congestion, nasal discharge (runny nose), nausea, vomiting, and diarrhea.

### Outcome Measures

Primary outcomes were development of grade 3 or higher TEAEs though day 28, SARS-CoV-2 RNA below the assay lower limit of quantification (LLoQ) (ie, <2.0 log_10_ copies/mL) from NP swabs at days 3, 7, 14, and time to improvement in symptoms. This was defined as the number of days from treatment start to the first of 2 consecutive days when all of the 13 targeted COVID-19 symptoms scored as moderate or severe at day 0 were scored as mild or absent and all targeted symptoms scored as mild or absent at day 0 were scored as absent.

Secondary outcomes included the composite of death or hospitalization from any cause through day 28, time to all 13 targeted symptoms absent for 4 consecutive days, and time to return to usual health for 2 and 4 consecutive days.

### Pharmacokinetic and Pharmacodynamic (PK/PD) Data Analyses

PK parameters included maximum concentration (Cmax), time of Cmax (Tmax), observed concentrations at 1, 3, 7, 14, and 28-days post-dose, and area under the concentration-time curve (AUC). The AUC from time 0 to infinity (AUC_0-∞_) calculated using sample collection times, was based on the statistical moment theory using the linear-up, log-down trapezoidal rule (Phoenix WinNonlin, version 8.3, Certara). Partial AUCs were calculated to hours 72, 168, 336, and 672 post infusion (AUC_0–72, 168, 336, or 672h_).

### Statistical Analysis

The planned sample size was 220 participants, with 110 receiving the BMS mAbs and 110 receiving pooled placebo. Assuming a non-evaluability rate of 10%, there would be greater than 80% power to detect a 20% absolute increase in the proportion of participants with unquantifiable SARS-CoV-2 RNA on a given evaluation day (days 3, 7, 14) using a 2-sided 5% type 1 error rate.

Analyses were based on a modified intention-to-treat (mITT) population that included all participants who were randomized to the BMS mAbs or the pooled placebo arm and started treatment. Participants were summarized based on their randomized treatment assignment.

The proportion of participants with SARS-CoV-2 RNA <LLoQ from NP swabs was compared between arms on days 3, 7, and 14 using a log-binomial model adjusted for day 0 log_10_-transformed SARS-CoV-2 RNA level that was fit using generalized estimating equations with robust variance estimation. The treatment eﬀect was summarized with a risk ratio (RR) and 95% confidence interval [CI] for each study visit and a joint Wald test across all 3 days.

The time to symptom improvement, time to symptom absence, and time to return to usual pre-COVID-19 health outcomes were estimated for each arm using Kaplan-Meier methods and compared between arms using the Gehan-Wilcoxon test. The proportion of participants with symptom progression was compared using a log-binomial model and summarized with a RR and 95% CI.

Kaplan-Meier estimates of cumulative probability of hospitalization or death through day 28 were calculated for each arm. The proportion of patients who were hospitalized or died due to any cause through day 28 was compared using Fisher's exact test.

The proportion of participants with grade 3 or higher TEAEs through day 28 was compared between arms using log-binomial regression and summarized with a RR and 95% CI. Serious adverse events (SAEs) and TEAEs of special interest were summarized descriptively.

Associations between PK parameters and participant outcomes were explored as post-hoc analyses. For associations with NP RNA shedding, the day 3 log-transformed concentrations of the BMS mAbs were used as these were obtained at approximately the same time as the day 3 NP swabs. The association between day 3 log_e_-transformed concentrations of BMS-986414 and BMS-986413 with change in SARS-CoV-2 RNA from NP swabs between day 0 and day 3 was assessed using an interval-censored parametric regression model with a normal distribution and adjusted for day 0 SARS-CoV-2 RNA levels [21]. Analyses were restricted to participants with quantifiable SARS-CoV-2 RNA levels at day 0 and a SARS-CoV-2 RNA result at day 3. Estimated mean changes (and 95% CIs) in SARS-CoV-2 RNA from NP swabs corresponding to a 1 log_e_ μg/mL increase in day 3 concentrations are reported.

For evaluation with symptom outcomes, the AUC_0-72h_ measure (excluding the 24-hour measurement) was used, as this time-based metric standardizes exposure across participants. The association between log_e_-transformed AUC_0-72h_ of BMS-986414 and BMS-986413 and the time to symptom improvement was assessed using Cox proportional hazards regression models. Hazard ratios (HR) and 95% CIs corresponding to a 1 log_e_ h*μg/mL increase in AUC_0-72h_ and *P* values from a Wald test are reported.

All tests were 2-sided with 5% type I error rate. Statistical analyses were conducted in SAS 9.4.

## RESULTS

### Characteristics of the Participants

A total of 224 participants were enrolled at 43 US sites between May 25 and August 12, 2021 (Supplemental Figure 1). With 13 participants excluded due to never starting treatment (n=6) or being from sites with data integrity concerns (n=7), there were 211 participants (105 BMS mAbs and 106 placebo) in the mITT analysis population. One participant who was randomized to placebo received the BMS mAbs.

The median age of participants was 40 years, 51% assigned female sex at birth, all participants were cis-gender, 86% identified as White and 9% as Black (Table 1). There was a modest imbalance in ethnicity between arms, with 51% Latinx in the BMS mAbs arm and 62% in the placebo arm. Median body mass index (BMI) was 27 kg/m^2^ and 34% reported symptom duration of > 5 days at time of enrollment. Overall, 41% received at least 1 dose of a COVID-19 vaccine. Seropositivity for anti-N or anti-S was 77 % in the BMS mAbs arm versus 62% in the placebo arm. Viral variant data were available for 147 participants, with 90% having the Delta variant.

**Table 1.** Baseline Characteristics

	BMS mAbs (n=105)	Placebo (n=106)	Total (n=211)
Age (years), median (quartiles)	42 (31, 52)	40 (29, 50)	40 (30, 52)
Age category, n (%)			
<60 years	90 (85.7)	97 (91.5)	187 (88.6)
≥60 years	15 (14.3)	9 (8.5)	24 (11.4)
Sex at birth, n (%)			
Male	48 (45.7)	55 (51.9)	103 (48.8)
Female	57 (54.3)	51 (48.1)	108 (51.2)
Gender			
Cis-Gender	105 (100)	106 (100)	211 (100)
Race, n (%)			
Asian	2 (1.9)	2 (1.9)	4 (1.9)
American Indian	1 (1.0)	0 (0)	1 (0.5)
Black/African American	10 (9.5)	10 (9.4)	20 (9.5)
Multiple	0 (0)	1 (0.9)	1 (0.5)
White	90 (85.7)	92 (86.7)	182 (86.3)
Other	2 (1.9)	1 (0.9)	3 (1.4)
Ethnicity, n (%)			
Hispanic or Latino	54 (51.4)	66 (62.3)	120 (56.9)
Not Hispanic or Latino	51 (48.6)	40 (37.7)	91 (43.1)
Country, n (%)			
USA	105 (100)	106 (100)	211 (100)
Baseline body mass index (kg/m^2^)^*^			
Median (quartiles)	27.1 (24.4, 31.1)	27.4 (23.8, 31.3)	27.4 (24.0, 31.3)
Days from symptom onset, n (%)^†^			
≤5	70 (66.7)	69 (65.1)	139 (65.9)
>5	35 (33.3)	37 (34.9)	72 (34.1)
Median (quartiles)	5.0 (3.0, 6.0)	5.0 (3.0, 6.0)	5.0 (3.0, 6.0)
At least one dose of SARS-CoV-2 Vaccination, n (%)			
Yes	46 (43.8)	41 (38.7)	87 (41.2)
No	59 (56.2)	65 (61.3)	124 (58.8)
NP Swab SARS-Co V-2 RNA, (n)	100	99	199
Not detected, n (%)	27 (27.0)	22 (22.2)	49 (24.6)
Detected, < LLoQ, n (%)	7 (7.0)	9 (9.1)	16 (8.0)
≥LLoQ, n (%)	66 (66.0)	68 (68.7)	134 (67.3)
NP Swab SARS-CoV-2 RNA (log_10_ copies/mL), (n)	100	99	199
Median (quartiles)	4.1 (0.7, 6.0)	4.9 (1.7, 6.3)	4.3 (1.7, 6.1)
Serology (Anti-N and Anti-S combined), n (%)	99	102	201
Negative^a^	23 (23.2)	39 (38.2)	62 (30.8)
Positive^b^	76 (76.8)	63 (61.8)	139 (69.2)
COVID variant, (n)	72	75	147
Delta, n (%)	68 (94.4)	64 (85.3)	132 (89.8)
Non-delta, n (%)^c^	4 (5.5)	11 (14.7)	15 (10.2)

BMS mAbs: subcutaneously administered BMS-986414 (C135-LS) 200 mg plus BMS-986413 (C144-LS) 200 mg; NP: Nasopharyngeal; LLoQ: Lower limit of quantification; Q1: lower quartile; Q3: upper quartile.

a Negative means negative for both Anti-N and Anti-S

b Positive means positive for either Anti-N or Anti-S

c Non Delta: Alpha, Gamma, Mu, Other

Symptoms reported by more than 50% of participants at enrollment included cough (84%), fatigue (81%), body or muscle pain (74%), stuﬀy nose (73%), headache (71%), runny nose (62%), chills (58%), feeling feverish (56%), and sore throat (56%) (Supplemental Figure 2).

At day 28, there were 93% in the BMS mAbs arm and 94% in the placebo arm who were still in the study. Thirteen participants (7 BMS mAbs and 6 placebo) discontinued study follow-up before or at day 28.

### Clinical Efficacy

**Figure 1.**
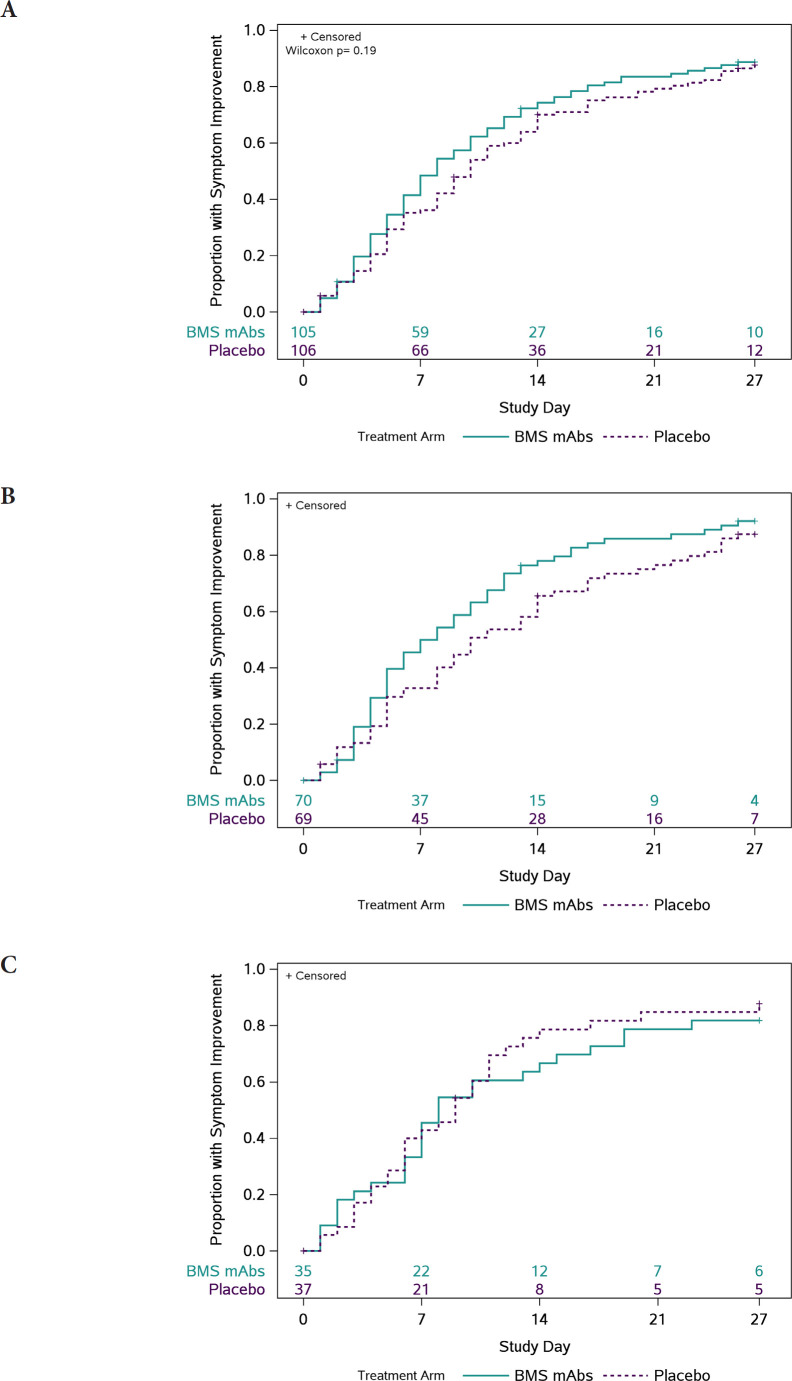
**Time (days) to all targeted symptoms improved from day 0 for 2 consecutive days, overall, and by days from symptom onset to enrollment.** The cumulative proportion of participants with all symptoms improved for 2 consecutive days was calculated using Kaplan-Meier methods for all participants (Panel A), participants who were randomized ≤ 5 days from symptom onset (Panel B), and randomized > 5 days from symptom onset (Panel C). Numbers above the x-axis indicates the number of participants still in follow-up who have not previously had 2 consecutive days with all targeted symptoms improved.

The distribution of times to symptom improvement favored the BMS mAbs arm (median 8 days [quartiles 4, 15]) relative to the placebo arm (10 days [5, 17]), but was not statistically significant (*P*=0.19) (Figure 1). A lower proportion of participants had worsening of ≥1 COVID-19 symptoms in the BMS mAbs arm versus the placebo arm (66.7% versus 80.2%; RR: 0.83 [95%CI: 0.70, 0.98]). There were no significant diﬀerences between arms with respect to other symptom-based outcomes, though time to return to usual health also favored the BMS mAbs arm (Supplemental Table 2). Three participants in the BMS mAbs arm had COVID pneumonia compared to 4 in the placebo arm (Supplemental Table 3).

There were 7 participants who were hospitalized through day 28, including 4 (3.8%) in the BMS mAbs arm and 3 (2.8%) in the placebo arm, with no deaths through study day 28.

### Virology

The proportion of participants with NP swab SARS-CoV-2 RNA levels below the LLoQ at day 0 was 34% in the BMS mAbs arm and 31% in the placebo arm (Table 2). The proportion with SARS-CoV-2 RNA below the LLoQ was similar at day 3 (47% in both arms), day 7 (79% vs 73%), and day 14 (91% vs 93%). Diﬀerences in the proportion below the LLoQ between arms (adjusted for day 0 levels) were not statistically significant at day 3 (RR: 1.03; 95%CI: 0.80, 1.32), day 7 (RR: 1.04; 95%CI: 0.94, 1.15), day 14 (RR: 1.00; 95%CI: 0.90, 1.12), or across time points (overall Wald test *P*=0.71).

**Table 2.** Nasopharyngeal Swab SARS-CoV-2 RNA by Visit and Treatment Arm

		BMS mAbs n=105	Placebo n=106	Analysis Results Risk Ratio [95%CI]
Day 0	n <LLoQ ≥LLoQ	100 34 (34.0%) 66 (66.0%)	99 31 (31.3%) 68 (68.7%)	
Day 3	n <LLoQ ≥LLoQ	89 42 (47.2%) 47 (52.8%)	93 44 (47.3%) 49 (52.7%)	1.03 [0.80, 1.32] Ref
Day 7	n <LLoQ ≥LLoQ	90 71 (78.9%) 19 (21.1%)	88 64 (72.7%) 24 (27.3%)	1.04 [0.94, 1.15] Ref
Day 14	n <LLoQ ≥LLoQ	90 82 (91.1%) 8 (8.9%)	91 85 (93.4%) 6 (6.6%)	1.00 [0.90, 1.12] Ref
Overall				*P*=0.71^a^

BMS mAbs, subcutaneously administered BMS-986414 (C135-LS) 200 mg plus BMS-986413 (C144-LS) 200 mg; LLoQ, lower limit of quantification of assay; NP, nasopharyngeal; CI, confidence interval Risk Ratio calculated as BMS mAbs versus placebo. Risk Ratio and 95% CI estimated from log-binomial models fitted using generalized estimating equations with an independent working correlation structure and robust standard errors.

a A joint test or randomized time points were assessed using a 2-sided Wald test.

### Safety

Fewer grade ≥3 TEAEs were reported in the BMS mAbs arm than in the placebo arm (7.6% versus 13.2%, respectively; RR: 0.58 [95%CI: 0.25, 1.32]) (Table 3). Drug-related TEAEs were uncommon, but higher in the BMS mAbs arm (4.8% vs 1.9%) and included single events of thrombocytopenia, hypersensitivity reaction, oropharyngeal pain, and increased creatinine and 3 injection reactions (one in the placebo arm). There was 1 reported TEAE of special interest, which was a hypersensitivity reaction in a BMS mAbs recipient. (Table 3).

**Table 3.** Safety Analysis through Day 28

	BMS mAbs n=105	Placebo n=106	Total n=211
Any TEAE, n (%)	38 (36.2)	36 (34.0)	74 (35.1)
Grade 3 or higher TEAE, n (%)	8 (7.6)	14 (13.2)	22 (10.4)
Serious TEAE, n (%)	4 (3.8)	3 (2.8)	7 (3.3)
Study drug related TEAE, n (%)	5 (4.8)	2 (1.9)	7 (3.3)
TEAE leading to study drug withdrawal, n (%)	0	0	0
TEAE with outcome of death, n (%)	0	0	0
TEAE of special interest^a^, n (%)	1 (1.0)	0	1 (0.5)

BMS mAbs refers to subcutaneously administered BMS-986414 (C135-LS) 200 mg plus BMS-986413 (C144-LS) 200 mg.

Treatment-emergent adverse events (TEAEs) are defined as adverse events new in onset or aggravated in severity or frequency from the baseline condition following the start of study treatment, including events reported as adverse events and graded laboratory value escalations.

a 1 participant had a Grade 1 hypersensitivity TEAE of special interest.

### Pharmacokinetic/Pharmacodynamic Data

There were 98 participants (93%) in the BMS arm with PK data available, including 37 with a concentration obtained on day 1. The median (quartiles) Tmax was 189 hours (163, 340) for BMS-986414 (C135-LS) and 162 hours (93, 169) for BMS-986413 (C144-LS). Observed median (quartiles) Cmax values were 17.6 µg/mL (13.8, 22.6) and 16.9 µg/mL (13.4, 23.6) for BMS-986414 and BMS-986413, respectively. Estimated median elimination half-life values (quartiles) were 80.0 days (70.4, 89.8) for BMS-986414 and 25.3 days (22.2, 28.9) for BMS-986413 (Supplemental Table 4).

**figure 2.**
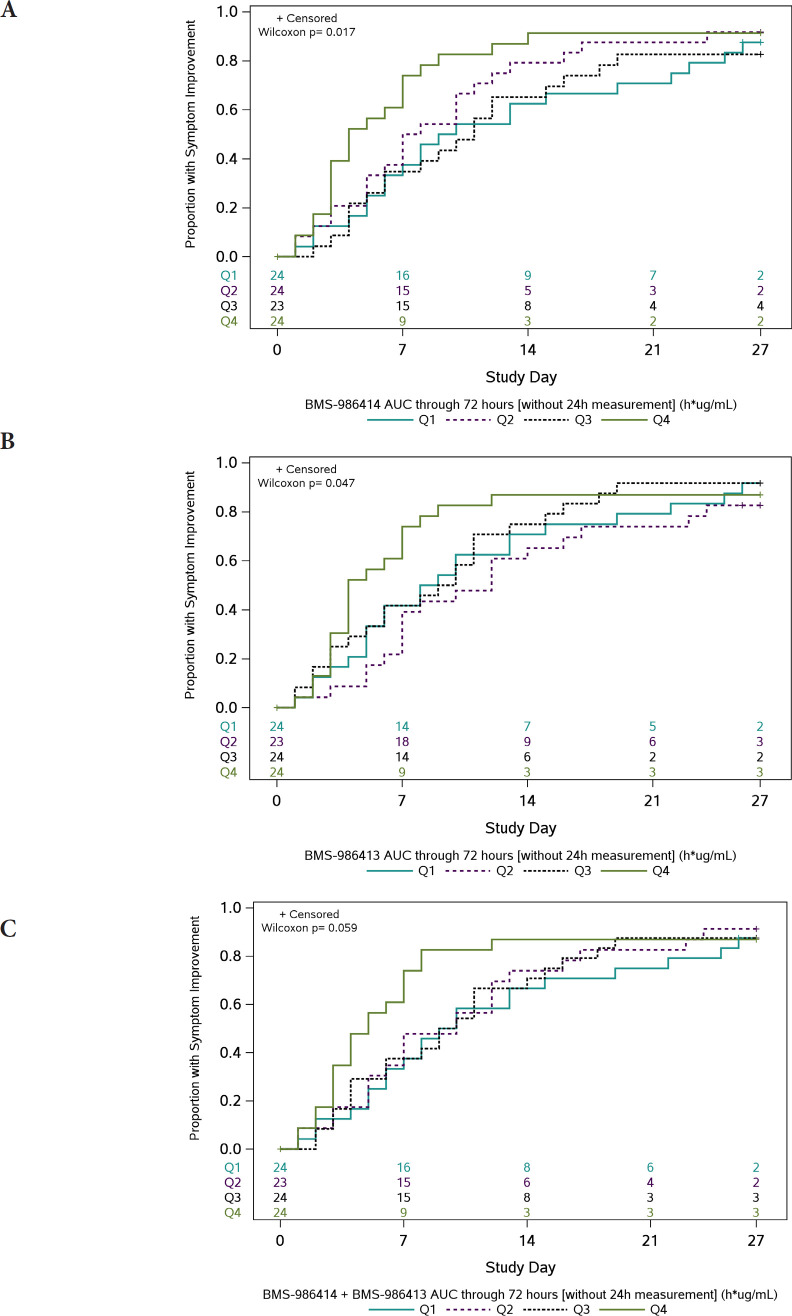
**Kaplan-Meier curves of time to symptom improvement (days) by quartiles of area under the concentration-time curve (AUC) through 72 hours.** The cumulative proportion of participants with all symptoms improved for 2 consecutive days was calculated using Kaplan-Meier methods for quartiles of AUC_0-72h_ (area under the concentration-time curve from time 0 to 72 hours) for (A) BMS-986414 (C135-LS), (B) BMS-986413 (C144-LS), and (C) BMS-986414 (C135-LS) + BMS-986413 (C144-LS). Numbers above the x-axis indicate the number of participants still in follow-up who have not previously had 2 consecutive days with all targeted symptoms improved. The time to symptom improvement was compared between quartiles using the Gehan-Wilcoxon test.

In post-hoc PD analyses, across 95 participants with an AUC_0-72h_ measure and symptom outcome, participants in the highest quartile of exposure had the fastest times to symptom improvement (Figure 2). There was an inverse trend between AUC_0-72h_ for BMS-986414 (C135-LS) and BMS-986413 (C144-LS) combined and time to symptom improvement, suggesting that participants with higher combined mAb AUC_0-72h_ had a shorter time to symptom improvement (HR 1.31 per log_e_ h^*^µg/mL higher combined AUC_0-72h_; 95% CI: 0.89, 1.90). When this relationship was examined by duration of symptoms at baseline, the trend was only apparent in the subgroup of participants with ≤5 days of symptoms, with an HR of 1.47 per 1 log_e_ h^*^µg/mL increase in combined mAb AUC_0–72h_ (95% CI: 0.96, 2.25) (Supplemental Table 5). When looking at each agent individually in this subgroup, the association was more pronounced with BMS-986414 (HR 1.57 per 1 log_e_ h^*^µg/mL increase; 95% CI: 1.01, 2.45) as compared to BMS-986413 (HR 1.37 per 1 log_e_ h^*^µg/mL increase; 95%CI: 0.92, 2.03).

For participants who had quantifiable NP RNA at baseline and a day 3 mAb concentration measure (N = 56), there was an inverse trend such that participants with higher combined day 3 mAb concentrations had, on average, a greater decrease in SARS-CoV-2 RNA levels from baseline to day 3 (mean diﬀerence: −0.73 log_10_ copies/mL (95% CI: −1.53, 0.07) per 1 log_e_ µg/mL higher day 3 mAb concentration). For BMS-986414 the mean diﬀerence was −0.91 log_10_ copies/mL (95% CI: −1.71, −0.12) per 1 log_e_ µg/mL higher day 3 concentration, and for BMS-986413 it was −0.54 log_10_ copies/mL (95% CI: −1.30, 0.23) per 1 log_e_µg/mL higher day 3 concentration (Supplemental Table 6).

## DISCUSSION

In this phase 2 study of BMS-986414 (C135-LS) and BMS-986413 (C144-LS), one of the first sub-cutaneous mAbs studied for the treatment of low-risk outpatient COVID-19 infection, the combination was safe but did not show a significant diﬀerence in time to symptom improvement compared to placebo. However, there was a trend favoring the active arm and participants on active treatment were less likely to have worsening symptoms. The primary virological endpoint was not significantly diﬀerent between the 2 arms. There were no deaths in the study and no significant diﬀerence in the small number of hospitalizations.

There are multiple potential contributors to why this combination mAb therapy, demonstrated to be highly active *in vitro* [12] including against the Delta variant (Christian Gaebler, MD, MSc, electronic communication, April 18, 2023), did not show a significant eﬀect on symptom improvement or NP viral shedding. First, the study only included a low-risk population due to the availability of emergency use-authorized IV mAbs for treatment of persons at higher risk for progression to severe COVID-19 (the population in which the clinical efficacy of IV mAbs was demonstrated), and there were no proven therapeutics for the low-risk population. Whether this antibody combination given SC would have efficacy in high-risk individuals is unknown. In addition, 41% had received partial or full vaccination against COVID-19 and 69% were seropositive for antibodies to SARS-CoV-2 at baseline, including a higher percentage of seropositivity in the BMS mAbs arm (77% BMS vs 62% placebo). This pre-existing immunity may have limited the eﬀect of mAb therapy. Finally, we now understand that viral shedding of SARS-CoV-2 decreases substantially in most people aﬅer 5 days without treatment [22], especially in vaccinated individuals [23]. More than one-third of our participants had symptoms of more than 5 days duration at enrollment, and at baseline 65 out of 211 of participants (31%) had NP viral loads that were not quantifiable. This high rate of participants below the LLoQ at baseline may have limited our ability to see diﬀerences between arms for primary and secondary virologic endpoints.

An unanswered question is whether adequate mAb concentrations were achieved fast enough at the site of infection to impact viral replication following SC administration. Approximately 24 hours aﬅer drug administration median plasma concentrations were 8.5 and 9.5 µg/mL for BMS-986414 and BMS-986413, respectively. Assuming 12% penetration into lung epithelial lining fluid (ELF) [24], predicted concentrations in ELF would be 1.02 and 1.14 µg/mL, respectively, for BMS-986414 and BMS-986413, suggesting that drug concentrations exceeded in *vitro* IC_90_ values (IC_90_ of 10.43 and 21.68 ng/mL respectively) by 100- and 52-fold [24]. However, in a study of a mAb targeting *Staphylococcus aureus* cytotoxins, the peak antibody concentrations in lung ELF were not achieved until an observed median of 192 hours aﬅer the dose, indicating a distinct lag phase for drug penetration from blood into ELF, which could raise uncertainty as to whether adequate concentrations with SC dosing were achieved fast enough [24]. We investigated relationships between concentrations achieved in the first 72 hours and efficacy as measured by NP RNA levels and symptom improvement. In the subset of participants with quantifiable NP RNA levels at baseline, higher combined and individual mAb concentrations at day 3 were associated with greater declines in NP RNA levels. Higher AUC_0-72h_ were also associated with shorter times to symptom improvement, and these associations were strongest in those with symptoms for 5 or fewer days. Antiviral response data of tixagevimab and cilgavimab administered intravenously versus intramuscularly in this platform study also support this observed relationship [16].

## CONCLUSION

There were several interesting lessons learned from this study. Our data suggest that an SC route of administration for a mAb might be a viable option for the treatment of COVID-19 when persons present early in the course of infection and are able to achieve early high concentrations of the mAb; use of higher doses may be needed to achieve adequate concentrations for clinical benefit. Our results inform the design of future studies of SC mAb administration viral respiratory pathogens. Continued exploration of the potential role of SC administration for mAbs is encouraged, not only to address purely logistical barriers to providing infusion therapy as has been the standard for SARS-CoV-2 mAb therapy, but importantly, to identify mechanisms to improve access to treatment and equitable treatment for vulnerable communities and communities of color. These deficiencies in our existing systems were demonstrated early in the pandemic [7–10]. Travel gap for patients in rural and underserved communities, provider shortages, as well as declination of EUA approved mAbs due to medical mistrust are among the many reasons vulnerable populations suffered disproportionally worse outcomes during the pandemic. Having SC administration provided in accessible locations has the potential to address some of these barriers, if an eﬀective anti-SARS-CoV-2 mAbs with activity against evolving variants can be developed. This study adds to the small, but important body of literature on clinical efficacy and pharmacokinetics of SC SARS-CoV-2 mAbs and informs SC mAb development for not only SARS-CoV-2 but also other viral infections.

## SUPPLEMENTARY DATA

Supplementary materials are available at the *Pathogens and Immunity* website. Supplementary data may be provided by the authors to benefit the reader. Supplementary data are not copyedited and are the sole responsibility of the authors. Questions or comments related to supplementary materials should be addressed to the corresponding author.
