# Supplementary material for: Safety, Efficacy, and Pharmacokinetics of Combination SARS-CoV-2 Neutralizing Monoclonal Antibodies BMS-986414 (C135-LS) and BMS-986413 (C144-LS) Administered Subcutaneously in Non-Hospitalized Persons with COVID-19 in a Phase 2 Trial
Source: Pathog Immun. 2024 May 6;9(1):138–55. doi: 10.20411/pai.v9i1.660 (PMC11093219; doi:10.20411/pai.v9i1.660)
Supplement: Supplementary Tables and Figures [file pai-9-138-s01.pdf]

PATHOGENS AND IMMUNITY
IN ASSOCIATION WITH CASE WESTERN RESERVE UNIVERSITY AND THE RICHARD J. FASENMYER FOUNDATION

SUPPLEMENTARY MATERIALS

Supplemental Table 1. Definition of Lower Risk of Progression of COVID-19 2

Supplemental Table 2. Analysis of COVID-19 Associated Symptoms Outcomes through Day 28 3

Supplemental Table 3. Grade 3 or higher Treatment-Emergent Adverse Events 4

Supplemental Table 4. Pharmacokinetic Characteristics by Monoclonal Antibody 5

Supplemental Table 5. Association between log_e-transformed $AUC_{0\text{-}72h}$ and Time to Symptom Improvement for 2 Consecutive Days 6

Supplemental Table 6: Association between log_e-transformed Concentration at Day 3 and Difference in Nasopharyngeal Swab SARS-CoV-2 RNA from Day 0 to Day 3 7

Supplemental Figure 1: Consort Diagram 8

Supplemental Figure 2: Baseline Symptoms by Severity 9
Participating Sites, Investigators, Study Personnel, and Additional Grant Support 10
ACTIV-2/A5401 Study Team 13

Corado KC, Chew KW, Giganti MJ, Mu Y, Fletcher CV, Currier JS, Daar ES, Wohl DA, Li JZ, Moser CB, Ritz J, Javan AC, Neytman G, Caskey M, Hughes MD, Smith DM, Eron JJ, ACTIV-2/A5401 Study Team. Safety, Efficacy, and Pharmacokinetics of Combination SARS-CoV-2 Neutralizing Monoclonal Antibodies BMS-986414 (C135-LS) and BMS-986413 (C144-LS) Administered Subcutaneously in Non-Hospitalized Persons with COVID-19 in a Phase 2 Trial. *Pathogens and Immunity*. 2024;9(1):138–155. doi: 10.20411/pai.v9i1.660

PATHOGENS AND IMMUNITY
IN ASSOCIATION WITH CASE WESTERN RESERVE UNIVERSITY AND THE RICHARD J. FASENMYER FOUNDATION

Supplemental Table 1: Definition of Lower Risk of Progression of COVID-19

**Lower risk to progression**	Not meeting the higher risk to progression definition
**Higher risk to progression**	Participants are considered at “higher” risk of progression to severe COVID-19 if they have at least one of the following factors:<br>• age 60 years and older and no history of SARS-CoV-2 vaccination<br>• any age with at least one of the following conditions (self-report is acceptable) and no history of SARS-CoV-2 vaccination:<br><br>1. current smoker (cigarette smoking within the past 30 days) AND history of at least 100 lifetime cigarettes<br>2. exogenous or endogenous immunosuppression defined as any of the following:<br>▪ HIV infection with CD4 count <200 cells/mm^3<br>▪ receiving corticosteroids equivalent to prednisone ≥20mg daily for at least 14 consecutive days within 30 days prior to study entry<br>▪ treatment with biologics (e.g., infliximab, abalizumab, ustekinumab, etc.), immunomodulators (e.g., methotrexate, 6MP, azathioprine, etc.), or cancer chemotherapy within 90 days prior to study entry<br><br>3. chronic lung disease or asthma requiring daily prescribed therapy<br>4. obesity (body mass index [BMI] >35; may be based on self-report of height and weight)<br>5. hypertension, with at least one medication recommended or prescribed<br>6. cardiovascular disease defined as history of any of the following: myocardial infarction, stroke, transient ischemic attack, heart failure, angina with prescribed nitroglycerin, coronary artery bypass grafts, percutaneous coronary intervention (PCI), carotid endarterectomy, and aortic bypass<br>7. diabetes mellitus<br>8. chronic kidney disease requiring hemodialysis or peritoneal dialysis<br>9. history of cirrhosis<br>10. active cancer, other than localized skin cancer

Corado KC, Chew KW, Giganti MJ, Mu Y, Fletcher CV, Currier JS, Daar ES, Wohl DA, Li JZ, Moser CB, Ritz J, Javan AC, Neytman G, Caskey M, Hughes MD, Smith DM, Eron JJ, ACTIV-2/A5401 Study Team. Safety, Efficacy, and Pharmacokinetics of Combination SARS-CoV-2 Neutralizing Monoclonal Antibodies BMS-986414 (C135-LS) and BMS-986413 (C144-LS) Administered Subcutaneously in Non-Hospitalized Persons with COVID-19 in a Phase 2 Trial. *Pathogens and Immunity*. 2024;9(1):138–155. doi: 10.20411/pai.v9i1.660

PATHOGENS AND IMMUNITY
In Association with Case Western Reserve University and The Richard J. Fasenmyer Foundation

Supplemental Table 2: Analysis of COVID-19 Associated Symptoms Outcomes through Day 28

	BMS mAbs (n=105)	Placebo (n=106)	Analysis Result
Time (days) to sustained symptom improvement for 2 consecutive days[a], median (quartiles)	8 (4, 15)	10 (5, 17)	p=0.19
Enrolled ≤5 days from symptom onset	8 (4, 13)	10 (5, 21)	
Enrolled >5 days from symptom onset	8 (6, 19)	9 (5, 13)	
Time (days) to all targeted symptoms absent for 4 consecutive days[a], median (quartiles)	13 (8, >25)	13 (8, 25)	p=0.69
Time (days) to return to usual pre-COVID-19 health for 2 consecutive days[a], median (quartiles)	12 (6, 25)	16 (8, >27)	p=0.17
Time (days) to return to usual pre-COVID-19 health for 4 consecutive days[a], median (quartiles)	13 (7, >25)	17 (8, >25)	p=0.08
Progression of one or more COVID-19 associated symptoms to a worse status than at day 0[b], n (%)	70 (66.7)	85 (80.2)	RR 0.83 [95%CI: 0.70, 0.98]

BMS mAbs refers to subcutaneously administered BMS-986414 (C135-LS) 200 mg plus BMS-986413 (C144-LS) 200 mg

[a] The time to symptom improvement, time to symptoms absent, and time to return to usual pre-COVID-19 health outcomes were each compared between arms using the Gehan-Wilcoxon test.

[b] The proportion of participants with symptom progression was compared between arms using log-binomial regression and summarized with a risk ratio (RR) and 95% confidence interval (95% CI).

Corado KC, Chew KW, Giganti MJ, Mu Y, Fletcher CV, Currier JS, Daar ES, Wohl DA, Li JZ, Moser CB, Ritz J, Javan AC, Neytman G, Caskey M, Hughes MD, Smith DM, Eron JJ, ACTIV-2/A5401 Study Team. Safety, Efficacy, and Pharmacokinetics of Combination SARS-CoV-2 Neutralizing Monoclonal Antibodies BMS-986414 (C135-LS) and BMS-986413 (C144-LS) Administered Subcutaneously in Non-Hospitalized Persons with COVID-19 in a Phase 2 Trial. *Pathogens and Immunity*. 2024;9(1):138–155. doi: 10.20411/pai.v9i1.660

PATHOGENS AND IMMUNITY
IN ASSOCIATION WITH CASE WESTERN RESERVE UNIVERSITY AND THE RICHARD J. FASENMYER FOUNDATION

Supplemental Table 3: Grade 3 or Higher Treatment-Emergent Adverse Events

Arm	ID	Adverse Event	Grade	Start day	End day	Related to Treatment
**BMS mAbs**	**1**	Blood creatinine increased	3	20	32	Related
	**2**	COVID-19 pneumonia	3	2	6	Not Related
	**3**	COVID-19 pneumonia	3	1	7	Not Related
	**4**	Suicide attempt	4	14	19	Not Related
	**5**	COVID-19 pneumonia	3	5	9	Not Related
	**6**	Lymphocyte count decreased	3	4	46	Not Related
	**7**	Headache	3	1	3	Not Related
	**8**	Blood glucose increased	3	14	498	Not Related
**Placebo**	**9**	Anxiety	3	10	35	Not Related
	**10**	COVID-19 pneumonia	3	8	13	Not Related
		Hypertransaminasaemia	3	9	44	Not Related
		Lymphopenia	4	3	28	Not Related
	**11**	Hyperglycaemia	3	3	32	Not Related
	**12**	COVID-19 pneumonia	3	0	12	Not Related
		Hypoxia	3	2	2	Not Related
	**13**	Blood bilirubin increased	3	20	503	Not Related
	**14**	COVID-19 pneumonia	3	3	7	Not Related
	**15**	COVID-19 pneumonia	3	3	9	Not Related
	**16**	Adverse drug reaction	4	15	35	Not Related
	**17**	Hypertension	3	7	252	Not Related
	**18**	Blood glucose increased	3	14	.	Not Related
	**19**	Alanine aminotransferase increased	3	14	30	Not Related
		Aspartate aminotransferase increased	3	2	30	Not Related
	**20**	Blood creatinine increased	4	14	31	Not Related
	**21**	Heavy menstrual bleeding	3	16	226	Not Related
	**22**	Anaemia	3	4	.	Not Related

BMS mAbs, subcutaneously administered BMS-986414 (C135-LS) 200 mg plus BMS-986413 (C144-LS) 200 mg. Each numbered ID reflects a unique participant (for several participants more than one grade 3 or higher adverse event was reported).

Corado KC, Chew KW, Giganti MJ, Mu Y, Fletcher CV, Currier JS, Daar ES, Wohl DA, Li JZ, Moser CB, Ritz J, Javan AC, Neytman G, Caskey M, Hughes MD, Smith DM, Eron JJ, ACTIV-2/A5401 Study Team. Safety, Efficacy, and Pharmacokinetics of Combination SARS-CoV-2 Neutralizing Monoclonal Antibodies BMS-986414 (C135-LS) and BMS-986413 (C144-LS) Administered Subcutaneously in Non-Hospitalized Persons with COVID-19 in a Phase 2 Trial. *Pathogens and Immunity*. 2024;9(1):138–155. doi: 10.20411/pai.v9i1.660

PATHOGENS AND IMMUNITY
IN ASSOCIATION WITH CASE WESTERN RESERVE UNIVERSITY AND THE RICHARD J. FASENMYER FOUNDATION

Supplemental Table 4: Pharmacokinetic Characteristics by Monoclonal Antibody

Variable	BMS-986414 (C135-LS)	BMS-986413 (C144-LS)
AUC_{0-72h} (h*µg/mL) Median (quartiles)	n=95<br>515 (302, 676)	n=95<br>555 (334, 813)
Concentration Day 1 (µg/mL) Median (quartiles)	n=37<br>8.5 (5.7, 10.8)	n=37<br>9.5 (7.7, 13.0)
Concentration Day 3 (µg/mL) Median (quartiles)	n=92<br>13.3 (9.5, 19.4)	n=92<br>15.3 (10.2, 23.4)
Cmax (µg/mL) Median (quartiles)	n=98<br>17.6 (13.8, 22.6)	n=98<br>16.9 (13.4, 23.6)
Tmax (h) Median (quartiles)	n=98<br>189 (163, 340)	n=98<br>162 (93, 169)
$AUC_{0-\infty}$ (h*µg/mL) Median (quartiles)	n=92<br>54927 (39067, 67168)	n=93<br>17445 (13594, 20900)
Half-Life (d) Median (quartiles)	n=92<br>80.0 (70.4, 89.8)	n=93<br>25.3 (22.2, 28.9)

Abbreviations: area under the concentration-time curve from time 0 to 72 hours, AUC_{0-72h}; maximum observed concentration, Cmax; time to maximum concentration, Tmax; area under the concentration-time curve from time 0 to infinity, $AUC_{0-\infty}$ (h*µg/mL); hours, h; days, d

Corado KC, Chew KW, Giganti MJ, Mu Y, Fletcher CV, Currier JS, Daar ES, Wohl DA, Li JZ, Moser CB, Ritz J, Javan AC, Neytman G, Caskey M, Hughes MD, Smith DM, Eron JJ, ACTIV-2/A5401 Study Team. Safety, Efficacy, and Pharmacokinetics of Combination SARS-CoV-2 Neutralizing Monoclonal Antibodies BMS-986414 (C135-LS) and BMS-986413 (C144-LS) Administered Subcutaneously in Non-Hospitalized Persons with COVID-19 in a Phase 2 Trial. *Pathogens and Immunity*. 2024;9(1):138–155. doi: 10.20411/pai.v9i1.660

PATHOGENS AND IMMUNITY
In Association with Case Western Reserve University and The Richard J. Fasenmyer Foundation

Supplemental Table 5: Association between $\log_e$-transformed $AUC_{0\text{-}72h}$ and Time to Symptom Improvement for 2 Consecutive Days

	BMS mAbs	Hazard Ratio (95% CI)	P-value
Overall (n=95)	BMS-986414	1.46 (0.99, 2.17)	0.057
	BMS-986413	1.18 (0.83, 1.68)	0.36
	BMS-986414+BMS-986413	1.31 (0.89, 1.90)	0.17
≤ 5 days from symptom onset (n=63)	BMS-986414	1.57 (1.01, 2.45)	0.045
	BMS-986413	1.37 (0.92, 2.03)	0.12
	BMS-986414+BMS-986413	1.47 (0.96, 2.25)	0.07
> 5 days from symptom onset (n=32)	BMS-986414	1.22 (0.57, 2.62)	0.60
	BMS-986413	0.81 (0.36, 1.79)	0.60
	BMS-986414+BMS-986413	0.98 (0.44, 2.17)	0.96

BMS mAbs, subcutaneously administered BMS-986414 (C135-LS) 200 mg plus BMS-986413 (C144-LS) 200 mg; $AUC_{0\text{-}72h}$: area under the concentration-time curve from time 0 to 72 hours; CI, confidence interval

Hazard ratios estimate the relative hazard of symptom improvement for 2 consecutive days corresponding to a 1 $\log_e$ h*µg/mL increase in $AUC_{0\text{-}72h}$. Hazard ratio estimates and corresponding 95% CIs were obtained from Cox proportional hazards regression models with an event outcome of time to 2 consecutive days with all targeted symptoms improved from day 0 status. A two-sided p-value from a Wald test is also reported.

Corado KC, Chew KW, Giganti MJ, Mu Y, Fletcher CV, Currier JS, Daar ES, Wohl DA, Li JZ, Moser CB, Ritz J, Javan AC, Neytman G, Caskey M, Hughes MD, Smith DM, Eron JJ, ACTIV-2/A5401 Study Team. Safety, Efficacy, and Pharmacokinetics of Combination SARS-CoV-2 Neutralizing Monoclonal Antibodies BMS-986414 (C135-LS) and BMS-986413 (C144-LS) Administered Subcutaneously in Non-Hospitalized Persons with COVID-19 in a Phase 2 Trial. *Pathogens and Immunity*. 2024;9(1):138–155. doi: 10.20411/pai.v9i1.660

PATHOGENS AND IMMUNITY
IN ASSOCIATION WITH CASE WESTERN RESERVE UNIVERSITY AND THE RICHARD J. FASENMYER FOUNDATION

Supplemental Table 6: Association between $\log_e$-transformed Concentration at Day 3 and Difference in Nasopharyngeal Swab SARS-CoV-2 RNA from Day 0 to Day 3

	BMS mAbs	Estimated incremental change (95% CI) in $\log_{10}$ SARS-CoV-2 RNA levels per 1 $\log_e$ µg/mL increase in Day 3 concentration	P-value
Overall (n=56)	BMS-986414	-0.91 (-1.71, -0.12)	0.024
	BMS-986413	-0.54 (-1.30, 0.23)	0.17
	BMS-986414+BMS-986413	-0.73 (-1.53, 0.07)	0.07
≤ 5 days from symptom onset (n=33)	BMS-986414	-0.79 (-1.82, 0.25)	0.14
	BMS-986413	-0.32 (-1.38, 0.75)	0.56
	BMS-986414+BMS-986413	-0.56 (-1.63, 0.52)	0.31
> 5 days from symptom onset (n=23)	BMS-986414	-1.08 (-2.40, 0.25)	0.11
	BMS-986413	-0.73 (-1.85, 0.39)	0.20
	BMS-986414+BMS-986413	-0.90 (-2.14, 0.33)	0.15

BMS mAbs, subcutaneously administered BMS-986414 (C135-LS) 200 mg plus BMS-986413 (C144-LS) 200 mg; CI, confidence interval; NP, Nasopharyngeal; LLOQ, lower limit of quantification of assay Analyses restricted to participants with day 0 NP SARS-CoV-2 RNA levels ≥ LLOQ. Estimates and corresponding 95% CIs were obtained from interval censored parametric regression models with a normal distribution and adjusted for day 0 NP SARS-CoV-2 RNA levels. A two-sided p-value from a Wald test is also reported.

Corado KC, Chew KW, Giganti MJ, Mu Y, Fletcher CV, Currier JS, Daar ES, Wohl DA, Li JZ, Moser CB, Ritz J, Javan AC, Neytman G, Caskey M, Hughes MD, Smith DM, Eron JJ, ACTIV-2/A5401 Study Team. Safety, Efficacy, and Pharmacokinetics of Combination SARS-CoV-2 Neutralizing Monoclonal Antibodies BMS-986414 (C135-LS) and BMS-986413 (C144-LS) Administered Subcutaneously in Non-Hospitalized Persons with COVID-19 in a Phase 2 Trial. *Pathogens and Immunity*. 2024;9(1):138–155. doi: 10.20411/pai.v9i1.660

PATHOGENS AND IMMUNITY
IN ASSOCIATION WITH CASE WESTERN RESERVE UNIVERSITY AND THE RICHARD J. FASENMYER FOUNDATION

Supplemental Figure 1: Consort Diagram

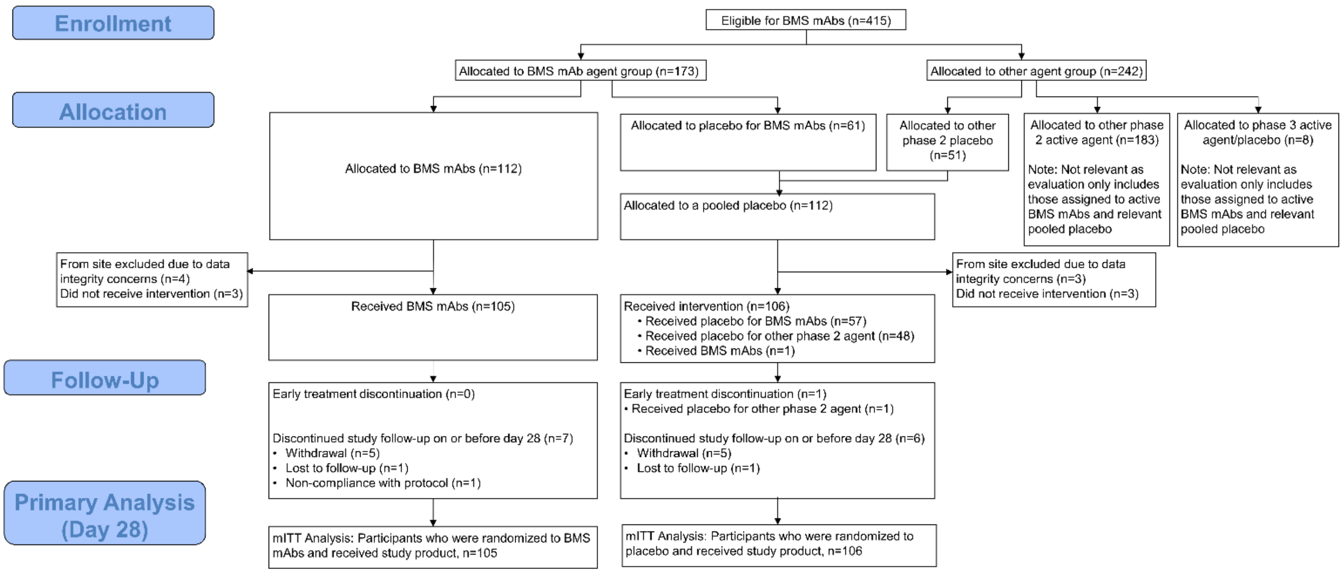

Corado KC, Chew KW, Giganti MJ, Mu Y, Fletcher CV, Currier JS, Daar ES, Wohl DA, Li JZ, Moser CB, Ritz J, Javan AC, Neytman G, Caskey M, Hughes MD, Smith DM, Eron JJ, ACTIV-2/A5401 Study Team. Safety, Efficacy, and Pharmacokinetics of Combination SARS-CoV-2 Neutralizing Monoclonal Antibodies BMS-986414 (C135-LS) and BMS-986413 (C144-LS) Administered Subcutaneously in Non-Hospitalized Persons with COVID-19 in a Phase 2 Trial. *Pathogens and Immunity*. 2024;9(1):138–155. doi: 10.20411/pai.v9i1.660

PATHOGENS AND IMMUNITY
In Association with Case Western Reserve University and The Richard J. Fasenmyer Foundation

Supplemental Figure 2: Baseline Symptoms by Severity

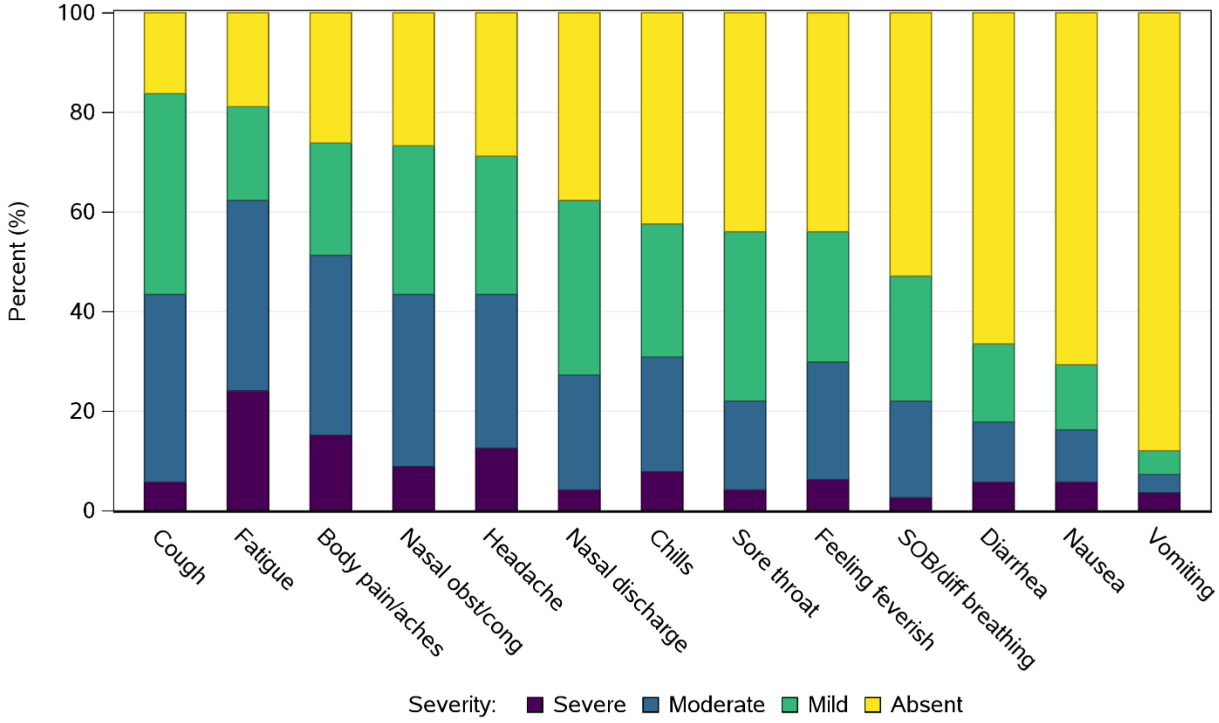

SOB: Shortness of Breath

Distribution of severity of 13 targeted symptoms on Day 0. Severity scored as severe (purple), moderate (blue), mild (green), or absent (yellow). Targeted symptoms are ordered by prevalence.

Corado KC, Chew KW, Giganti MJ, Mu Y, Fletcher CV, Currier JS, Daar ES, Wohl DA, Li JZ, Moser CB, Ritz J, Javan AC, Neytman G, Caskey M, Hughes MD, Smith DM, Eron JJ, ACTIV-2/A5401 Study Team. Safety, Efficacy, and Pharmacokinetics of Combination SARS-CoV-2 Neutralizing Monoclonal Antibodies BMS-986414 (C135-LS) and BMS-986413 (C144-LS) Administered Subcutaneously in Non-Hospitalized Persons with COVID-19 in a Phase 2 Trial. *Pathogens and Immunity*. 2024;9(1):138–155. doi: 10.20411/pai.v9i1.660

PATHOGENS AND IMMUNITY
IN ASSOCIATION WITH CASE WESTERN RESERVE UNIVERSITY AND THE RICHARD J. FASENMYER FOUNDATION

Participating Sites, Investigators, Study Personnel, and Additional Grant Support

Advance Medical Research Center, Miami, FL, USA: Ana Acosta (PI), Yenis Barbachan, Madelyn Ara

Allianz Research Institute Inc, Westminster, CA, USA: David N. Pham (PI), Wei-Hsin Kao

Ascension St. John Clinical Research Institute, Tulsa, OK, USA: Anju Malik (PI), Anna Bryan, Melanie Arnold

Best Quality Research, Inc, Hialeah, FL, USA, USA: Idania Fernandez (PI), Cinzia Karpf, Aniuska Ruiz

Bradenton Research Center Inc, Bradenton, FL, USA: Eric Folkens (PI)

Case Western Reserve University, Cleveland, OH, USA: Jeffrey Jacobson (PI), Leila Hojat, Julie Pasternak; additional grant support: UM1AI069501

Clinical Research Partners LLC, Richmond, VA, USA: Robert Call (PI), Leroy Vaughan

Clinical Trials Center of Middle Tennessee, Franklin, TN, USA: Aaron Milstone (PI), Jamie Alex Slandzicki, Jessica Wallan

Cullman Clinical Trials, Cullman, AL, USA: Randall Quinn (PI)

Doctors Hospital at Renaissance Health Institute for Research and Development, Edinburg, TX, USA: Marissa Gomez-Martinez (PI), Luis Cantu, Monica Betancourt-Garcia

Fairway Medical Clinic, Houston, TX, USA: Murtaza Mussaji (PI)

Gonzalez MD & Aswad MD Health Services, Miami, FL, USA: Yaneicy Gonzalez Rojas (PI), Ahmad Aswad

Harbor UCLA Clinical Research Site, Torrance, CA, USA: Eric Daar (PI), Sadia Shaik, Timothy Hatlen; additional grant support: UM1A1069424, UL1TR000124

IACT Health – Roswell – IACT – HyperCore, Columbus, GA, USA: Joseph Surber (PI), Jeffrey Kingsley, April Pixler

Imagine Research of Palm Beach County, Boynton Beach, FL, USA: Alex Zopo (PI), Jack Herman, Craig Herman

Corado KC, Chew KW, Giganti MJ, Mu Y, Fletcher CV, Currier JS, Daar ES, Wohl DA, Li JZ, Moser CB, Ritz J, Javan AC, Neytman G, Caskey M, Hughes MD, Smith DM, Eron JJ, ACTIV-2/A5401 Study Team. Safety, Efficacy, and Pharmacokinetics of Combination SARS-CoV-2 Neutralizing Monoclonal Antibodies BMS-986414 (C135-LS) and BMS-986413 (C144-LS) Administered Subcutaneously in Non-Hospitalized Persons with COVID-19 in a Phase 2 Trial. *Pathogens and Immunity*. 2024;9(1):138–155. doi: 10.20411/pai.v9i1.660

Innovative Health Medical Center, Hollywood, FL, USA: Fernando Gonzalez Vergara (PI), Ana I. Gonzalez, Noemi Gonzalez

International Medical Investigational Center (IMIC) Inc., Palmetto Bay, FL, USA: Ramon Leon (PI), Boris Nikolov

James J Peters VA Medical Center - (CRS) – NAVREF, Bronx, NY, USA: Michael Gelman (PI), Olga Andriunas, Zarema Jagizarov; additional grant support: NIAID CRADA

Lakes Research, Miami Lakes, FL, USA: Jose Pérez (PI), Eloy Roman, Heriberto Fernández

Las Vegas Medical Research, Las Vegas, NV, USA: Bharat Mocherla (PI), Kelly Beck, Valarie Maldonado

Miami Clinical Research, Miami, FL, USA: Keila Hoover (PI)

Moore Clinical Research, Inc. - Brandon – HyperCore, Brandon, FL, USA: George W. Monlux, Jr (PI), Elizabeth Juneja, Arthur Wernick

Northwestern University, Chicago, IL, USA: Babafemi O Taiwo (PI), Claudia Hawkins, Baiba Berzins; additional grant support: UM1AI069471, UM1AI068636-14S2, UL1TR001422

Orlando Immunology Center Clinical Research Site, Orlando, FL, USA: Edwin DeJesus (PI), Charlotte-Paige Rolle

PPD Virtual - Science 37, Inc, Culver City, CA, USA: Debra Weinstein (PI)

Pro Live Medical Research Corp, Miami, FL, USA: Rosa M. Suarez (PI), Ezequiel Socorro, Estefania Socorro

Quantum Clinical Trials, Miami Beach, FL, USA: Gene Neytman (PI), Jack Herman, Craig Herman

Stanford University, Palo Alto, CA, USA: Upinder Singh (PI), Prasanna Jagannathan (PI), Divya Pathak

STAT Research, Springboro, OH, USA: Joshua J. Ordway (PI), Megan Heffner

Synergy Healthcare, Bradenton, FL, USA: Patrick Weston (PI), Khalilah Weston

Triple O Research Institute PA, West Palm Beach, FL, USA: Olayemi Osiyemi (PI), Myriam Izquierdo, Odelsey Torna

University of California Los Angeles, Los Angeles, CA, USA: Kara Chew (PI), Aleen Khodabakhshian, Samantha Fortier; additional grant support: UM1AI69424, UL1TR001881

University of California San Diego Antiviral Research Center, San Diego, CA, USA: Constance Benson (PI), Steven Hendrickx, Rosemarie Ramirez

University of California San Francisco, San Francisco, CA, USA: Anne Luetkemeyer (PI), Suzanne Hendler, Dennis Dentoni-Lasofsky; additional grant support: UM1AI069496, UL1TR001872

University of North Carolina at Chapel Hill, Chapel Hill, NC, USA: David Wohl (PI), Jonathan Oakes, Amy James Loftis; additional grant support: UM1AI069423, UL1TR002489

UT Southwestern HIV/ID Clinical Trials Unit, Dallas, TX, USA: Mamta K. Jain (PI), Smruthi Senthil, Kimberly Turner-Gray

Vida Clinical Studies, Dearborn, MI, USA: Derrick Williamson (PI), Hisham Atriss, Matthew Caloura

PATHOGENS AND IMMUNITY
In Association with Case Western Reserve University and The Richard J. Fasenmyer Foundation

ACTIV-2/A5401 Study Team

Kara Chew, MD, MS, Co-Chair, David Geffen School of Medicine at University of California, Los Angeles, Los Angeles, CA, USA

David (Davey) Smith, MD, MAS, Co-Chair, University of California, San Diego, La Jolla, CA, USA

Eric Daar, MD, Vice Chair, Lundquist Institute at Harbor-UCLA Medical Center, Torrance, CA, USA

David Wohl, MD, Vice Chair, University of North Carolina at Chapel Hill School of Medicine, Chapel Hill NC, USA

Judith Currier, MD, MSc, Protocol Investigator and ACTG Chair, David Geffen School of Medicine at University of California, Los Angeles, Los Angeles, CA, USA

Joseph Eron, MD, Protocol Investigator and ACTG Vice Chair, University of North Carolina at Chapel Hill School of Medicine, Chapel Hill NC, USA

Arzhang Cyrus Javan, MD, MPH, DTM&H, NIH Division of AIDS (DAIDS) Clinical Representative, National Institutes of Health, Rockville, MD, USA

Michael Hughes, PhD, Lead Statistician, Harvard T.H. Chan School of Public Health, Boston, MA, USA

Carlee Moser, PhD, Statistician, Harvard T.H. Chan School of Public Health, Boston, MA, USA

Mark Giganti, PhD, Statistician, Harvard T.H. Chan School of Public Health, Boston, MA, USA

Justin Ritz, MS, Statistician, Harvard T.H. Chan School of Public Health, Boston, MA, USA

Lara Hosey, MA, Clinical Trials Specialist, AIDS Clinical Trials Group (ACTG) Network Coordinating Center, Social & Scientific Systems, a DLH Company, Silver Spring, MD, USA

Jhoanna Roa, MD, Clinical Trials Specialist, AIDS Clinical Trials Group (ACTG) Network Coordinating Center, Social & Scientific Systems, a DLH Company, Silver Spring, MD, USA

Nilam Patel, Clinical Trials Specialist, AIDS Clinical Trials Group (ACTG) Network Coordinating Center, Social & Scientific Systems, a DLH Company, Silver Spring, MD, USA

Kelly Colsh, PharmD, DAIDS Pharmacist, NIH/DAIDS Pharmaceutical Affairs Branch, Rockville, MD, USA

Irene Rwakazina, PharmD, DAIDS Pharmacist, NIH/DAIDS Pharmaceutical Affairs Branch, Rockville, MD, USA

Justine Beck, PharmD, DAIDS Pharmacist, NIH/DAIDS Pharmaceutical Affairs Branch, Rockville, MD, USA

Scott Sieg, PhD, Protocol Immunologist, Case Western Reserve University, Cleveland, OH, USA

Jonathan Li, MD, MMSc, Protocol Virologist, Brigham and Women's Hospital, Harvard Medical School, Boston, MA, USA

Courtney Fletcher, PharmD, Protocol Pharmacologist, University of Nebraska Medical Center, Omaha, NE, USA

William Fischer MD, Protocol Critical Care Specialist, University of North Carolina at Chapel Hill School of Medicine, Chapel Hill NC, USA

Teresa Evering, MD, MS, Protocol Investigator, Weill Cornell Medicine, New York, NY, USA
Rachel Bender Ignacio, MD, MPH, Protocol Investigator, University of Washington, Seattle, WA, USA

Sandra Cardoso, MD, PhD, Protocol Investigator, Fundação Oswaldo Cruz, Rio de Janeiro, Brazil

Katya Corado, MD, Lundquist Institute at Harbor-UCLA Medical Center, Torrance, CA, USA

Prasanna Jagannathan, MD, Protocol Investigator, Stanford University School of Medicine, Palo Alto, CA, USA

Nikolaus Jilg, MD, PhD, Protocol Investigator, Massachusetts General Hospital, Harvard Medical School, Boston, MA, USA

Alan Perelson, PhD, Protocol Investigator, Los Alamos National Laboratory, Los Alamos, NM, USA

PATHOGENS AND IMMUNITY
In Association with Case Western Reserve University and The Richard J. Fasenmyer Foundation

Sandy Pillay, MB, CHB, Protocol Investigator, Enhancing Care Foundation, Durban, KwaZulu-Natal, South Africa

Cynthia Riviere, MD, Protocol Investigator, GHESKIO Center, Port-au-Prince, Haiti
Upinder Singh, MD, Protocol Investigator, Stanford University School of Medicine, Palo Alto, CA, USA

Babafemi Taiwo, MBBS, MD, Protocol Investigator, Northwestern University Feinberg School of Medicine, Chicago, IL, USA

Joan Gottesman, BSN, RN, CCRP, Field Representative, Vanderbilt University Medical Center, Nashville, TN, USA

Matthew Newell, BSN, RN, CCRN, Field Representative, University of North Carolina at Chapel Hill School of Medicine, Chapel Hill NC, USA

Susan Pedersen, BSN, RN, Field Representative, University of North Carolina at Chapel Hill School of Medicine, Chapel Hill NC, USA

Joan Dragavon, MLM, Laboratory Technologist, University of Washington, Seattle, WA, USA

Cheryl Jennings, BS, Laboratory Technologist, Northwestern University, Chicago, IL, USA

Brian Greenfelder, BA, Laboratory Technologist, Ohio State University, Columbus, OH, USA

William Murtaugh, MPH, Laboratory Specialist, ACTG Laboratory Center, University of California, Los Angeles, Los Angeles, CA, USA

Jan Kosmyna, MIS, RN, CCPR, ACTG Community Scientific Subcommittee Representative, Case Western University Clinical Research Site, North Royalton, OH, USA

Morgan Gapara, MPH, International Site Specialist, ACTG Network Coordinating Center, Social & Scientific Systems, a DLH Company, Durham, NC, USA

Akbar Shahkolahi, PhD, International Site Specialist, ACTG Network Coordinating Center, Social & Scientific Systems, a DLH Company, Silver Spring, MD, USA
